# 

**Published:** 1874-04

**Authors:** 


☞ Write your names, Post Office, County and State *plainly.* Be sure to say what Post Office you wish the RECORD sent.

Send money by check or Postal Order. Where these cannot be obtained, send at our risk, by registered letters.

☞ All communications, including remittances of money, etc., must be addressed to POWELL & GOLDSMITH.

MANY of our subscribers have secured premiums, all of which have been forwarded. We thank these friends for the exertions they have put forth for extending our circulation.

THE Pocket Formulary is still offered as a premium to all sending two new subscribers. Those sending names will do us the favor to claim the premiums by letter.

DR. T. CURTIS SMITH, of Ohio, is awarded the Electro-Magnetic Machine—having secured the largest number of subscribers over ten. Thank you Doctor.

ALL who are entitled to Wood's Household Magazine and Chromo and have not yet received them, will please write to that Magazine, at Newburg, N. Y. Their names, address and money have been forwarded.

IF ANY mistakes, etc., should occur in the Business Department we will take the greatest pleasure in attending to, or placing things right. If any one is neglected or overlooked in any particular they will do us the favor to notify us, and their wishes shall be attended to promptly. Please remember this.

### ASSOCIATE EDITORS:

GEORGIA.

James A. Long, M.D.

W. L. M. Harris, M.D.

H. L Battle, M.D.

W. C. Musgrove, M D

L B. Bouchelle, M.D.

Thomas Burdell, M.D.

E. H. W. Hunter, M.D.

V. S Hitt, M.D.

J. E. Pope, M.D.

A. H. Brantley, M.D.

J. J. Knott, M.D.

J. C. Wisdom, M.D.

W. W. Leake, M.D.

S. G. WHITE, M.D.

W. H. HALL, M.D.

G. D. CASE, M.D.

KENTUCKY.

W. S. Taylor, M.D.

B. W. Stone, M.D.

Charles Kearns, M.D.

TEXAS.

S. M. Welch, M.D.

T. J. Heard, M.D.

W. A. Heard, M.D.

TENNESSEE.

J. D. Tucker, M.D.

ALABAMA.

J. C. Knox, M.D.

Geo. F. Taylor, M.D.

J. B. Barnett, M.D.

S. M. Hogan, M.D.

D. S. Hopping, M.D.

J. T. Searcy, M.D.

S. F. Hill, M.D.

NORTH CAROLINA.

J. R. Hughes, M.D.

S. S. Satchwell, M.D.

A. B. Pierce, M.D.

H. Kelly, M.D.

Geo. A. Foote, M.D.

E. A. Anderson, M.D

H. T. Bahnson, M.D.

Willis Alston, M.D.

Thos. F. Wood, M.D.

N. J. Pittman, M.D.

John W. Booth, M.D.

SOUTH CAROLINA.

E. T. McSwain, M.D.

G. M. Rivers, M.D.

OHIO.

J. Q. A. Hudson, M.D.

C. H. Tidd, M.D.

VIRGINIA.

John H. Claiborne, M.D.

F. Horner, Jr., M.D.

R. J. Preston, M.D,

J. E. Lockridge, M.D.

J. S. Wharton, M.D.

Wm. O. Owen, M.D.

Sam'l P. Ripley, M.D.

Benj. Blackford, M.D.

E. A. Drewry, M.D.

Rob B. Tunstall, M.D.

W. F. Barr, M.D.

L. B. Anderson, M.D.

J. M. Lazzell, M.D.

MISSISSIPPI.

C. B. Gallaway, M.D,

Edward Lea, M.D.

S. V. D. Hill, M.D.

E. T. Henry, M.D.

T. P. Lockwood, M.D.

Robert Kells, M.D.

ARKANSAS.

A. D. Hodge, M.D.

J. K. Hodge, M.D.

A. W. Hobson, M.D.

NEW JERSEY.

J. B. MATTISON, M.D.

### CORRESPONDING EDITORS:

Ely Van DeWarker, M.D., New York.

C. C. F. Gay, M.D., New York, *Surgeon of Buffalo General Hospital.*

M. H. Henry, M.D., New York, *Surgeon to N. Y. Dispensary, Dep't of Venerial and Skin Diseases.*

Benjamin Howard, M.D., New York.

James Tyson, M.D., Philadelphia, Pa.

W. W. Keen, M.D. Philadelphia.

Geo. Strawbridge, M.D., Philadelphia.

Robert Robson, M D., Indiana.

A. Patton, M.D., Indiana.

John H. Weir, M.D., Illinois.

Thomas J Gallaher, M. D, Pennsylvania.

S. C. Busey, M.D., Washington, D. C.

Wm. R. Whitehead, M.D., Colorado Territory.

### LIST OF CASH PAYMENTS TO THE RECORD:

For 1873.

| | |
|---|---|
| Dr J N Gilmore, Alabama | $2 50 |
| Dr S G Bittick, Texas | 2 50 |
| Dr R D Carver, Alabama | 2 50 |
| Dr S L Bonner, Alabama | 2 50 |
| Dr J B Barnett, Alabama | 2 50 |
| Dr E A Rowan, Mississippi | 2 50 |
| Dr S V D Hill, Mississippi | 2 50 |
| Dr J D Moore, Georgia | 2 50 |
| Dr John D Tucker, Tennessee | 2 50 |
| Dr G McDonald, West Va | 2 50 |
| Dr W W Leak, Georgia | 2 50 |
| Dr J W Drewry, N. C | 2 50 |

For 1874.

| | |
|---|---|
| Dr W A Cusick, Oregon | 2 50 |
| Dr Thomas L Kerr, Texas | 2 50 |
| Dr J C Hanna, Arkansas | 2 50 |
| Dr B A Carl, Arkansas | 2 50 |
| Dr R D Carver, Alabama | 2 50 |
| Dr Thomas H Tarry, Tennessee | 2 50 |
| Dr L M Lovelace, Kentucky | 2 50 |
| Dr W D Hunt, Kentucky | 2 50 |
| Dr Benj. F Rea, Alabama | 2 50 |
| Dr J T C Seay, Alabama | 2 50 |
| Dr John T Dixon, Georgia | 2 50 |
| Dr A J Lutherloh, N. C | 2 50 |
| Dr E A Rowan, Mississippi | 2 50 |
| Dr R M Tucker, Alabama | 2 50 |
| Dr J Y Henderson, Mississippi | 2 50 |
| Dr P Taylor, Mississippi | 2 50 |
| Dr J Stewart & Son, Georgia | 2 50 |

For 1874.

| | |
|---|---|
| Dr W C Musgrove, Georgia | 2 50 |
| Dr J W Anthony, Georgia | 2 50 |
| Dr B L Payne, Ohio | 2 50 |
| Dr E H Trickle, Ohio | 2 50 |
| Dr George Eberhart, Georgia | 2 50 |
| Dr M V Harrington, Arkansas | 2 50 |
| Dr R D Lucius, Mississippi | 2 50 |
| Dr C R Smith, Mississippi | 2 50 |
| Dr W F Jorden, Alabama | 2 50 |
| Dr J G Smythe, Mississippi | 2 50 |
| Dr J D Moore, Georgia | 2 50 |
| Dr J W Butts, Georgia | 2 50 |
| Dr E H M Parham, Arkansas | 2 50 |
| Dr J A Ashford, Mississippi | 2 50 |
| Dr C McGuffey, Texas | 2 50 |
| Dr Robt. McDaniel, S. C | 2 50 |
| Dr J W Wimbish, La | 2 50 |
| Dr L W Coleman, N. C | 2 50 |
| Dr D R Fox, La | 2 50 |
| Dr James O Fox, Arkansas | 2 50 |
| Drs Paschall & Young, N. C | 2 50 |
| Dr W S Sheppard, S. C | 2 50 |
| Dr M G Williams, Georgia | 2 50 |
| Dr M P Deadwyler, Georgia | 2 50 |
| Dr A J Mathews, Georgia | 2 50 |
| Dr John W Starr, Georgia | 2 50 |
| Dr J L Hill, Oregon | 2 50 |
| Dr John R Wilson, N. C | 2 50 |
| Dr William H Wilson, Georgia | 2 50 |
| Dr M W Hill, N. C | 2 50 |
| Dr L A Grizzard, Texas | 2 50 |
| Dr M D Liles, Alabama | 2 50 |
| Dr R H Baylor, Va | 2 50 |

The above list of names are those who have paid for volume '73 and '74. If we have failed to credit any, they will do us the favor to inform us of the fact. April, 1874.

